# CT-derived sarcopenia should not preclude surgical stabilization of traumatic rib fractures

**DOI:** 10.1186/s41747-021-00206-4

**Published:** 2021-02-16

**Authors:** Derrick A. Doolittle, Matthew C. Hernandez, Francis I. Baffour, Michael R. Moynagh, Naoki Takahashi, Adam T. Froemming, Katrina N. Glazebrook, Brian D. Kim

**Affiliations:** 1 Department of Radiology, Mayo Clinic, 200 First Street SW, Rochester, MN 55905 USA; 2 Department of Surgery, Mayo Clinic, 200 First Street SW, Rochester, MN 55905 USA

**Keywords:** Length of stay, Muscle (skeletal), Rib fractures, Sarcopenia, Tomography (x-ray computed)

## Abstract

**Background:**

Rib fractures are associated with considerable morbidity and mortality. Surgical stabilization of rib fractures (SSRF) can be performed to mitigate complications. Sarcopenia is in general known to be associated with poor clinical outcomes. We investigated if sarcopenia impacted number of days of mechanical ventilation, intensive care unit (ICU) stay, and total hospital stay in patients who underwent SSRF.

**Methods:**

A retrospective single institutional review was performed including patients who underwent SSRF (2009–2017). Skeletal muscle index (SMI) was semiautomatically calculated at the L3 spinal level on computed tomography (CT) images and normalized by patient height. Sarcopenia was defined as SMI < 55 cm^2^/m^2^ in males and < 39 cm^2^/m^2^ in females. Demographics, operative details, and postoperative outcomes were reviewed. Univariate and multivariate analyses were performed.

**Results:**

Of 238 patients, 88 (36.9%) had sarcopenia. There was no significant difference in number of days of mechanical ventilation (2.8 ± 4.9 *versus* 3.1 ± 4.3, *p* = 0.304), ICU stay (5.9 ± 6.5 *versus* 4.9 ± 5.7 days, *p* = 0.146), or total hospital stay (13.3 ± 7.2 *versus* 12.9 ± 8.2 days, *p* = 0.183) between sarcopenic and nonsarcopenic patients. Sarcopenic patients demonstrated increased modified frailty index scores (1.5 ± 1.1 *versus* 0.9 ± 0.9, *p <* 0.001) compared to nonsarcopenic patients.

**Conclusions:**

For patients who underwent SSRF for rib fractures, sarcopenia did not increase the number of days of mechanical ventilation, ICU stay, or total hospital stay. Sarcopenia should not preclude the utilization of SSRF in these patients.

## Key points

- Sarcopenia is a general risk factor for poor clinical outcomes.
- Sarcopenic and non-sarcopenic patients who underwent surgical stabilization of traumatic rib fractures were compared.
- Sarcopenic patients did not have greater number of days of mechanical ventilation, intensive care unit stay, or total hospital stay.

## Background

Rib fractures are common following chest wall trauma and result in considerable morbidity and mortality, especially in those with diminished physiologic and pulmonary reserve [1]. Mortality after rib fractures is approximately 10% and increasing numbers of fractured ribs is associated with greater mortality and morbidity rates [2, 3]. In an effort to reduce morbidity and mortality associated with traumatic rib fractures, several investigators have shown benefits of surgical stabilization of rib fractures (SSRF), which include reductions in mechanical ventilation duration, mortality rates, development of pneumonia, pain, and long-term disability [4–8].

Sarcopenia is defined as the loss of skeletal muscle mass and function [9]. Multiple studies in a number of clinical and surgical scenarios have demonstrated that sarcopenic patients have worse outcomes than nonsarcopenic patients [9–21]. Furthermore, in patients with traumatic injuries, sarcopenia has been associated with increased mortality rates, duration of hospital stay, risk of complications, and more frequent patient discharges to a dependent facility [22, 23].

In this study, we aimed at evaluating the impact of sarcopenia in patients who underwent SSRF for traumatic rib fractures, hypothesizing that the presence of sarcopenia would impact short-term outcomes such as duration of mechanical ventilation, intensive care unit (ICU) stay, and total hospital stay.

## Methods

### Patient cohort

Institutional review board approval was obtained for this study, which was in compliance with the Health Insurance Portability and Accountability Act. A single institution retrospective review of patients who underwent SSRF for traumatic rib fractures from 2009 to 2017 at an American College of Surgeons (ACS) verified level I trauma center was performed. Institutional guidelines for SSRF include the following: flail chest; rib displacement greater than or equal to one rib width; clinically relevant and refractory pain in association with rib fracture(s); mobile rib fracture(s), *i.e.*, “clicking”; and anticipated nonunion or malunion of fractures. Exclusion criteria included patients who did not consent for research and those that did not have an admission computed tomography (CT) scan that included the L3 vertebral body level.

### CT scanning technique

Most exams were performed as part of an initial trauma evaluation and scanned on either a Siemens Flash scanner or Siemens Definition Edge scanner. Standard CT parameters for the trauma protocol were 2.0 mm slice thickness with 1.2 mm slice increment for the chest, with both a vascular optimized kernel (B30f) axial reconstruction and a dedicated high spatial resolution kernel for fracture evaluation (B70). The abdominopelvic portion of the exam was reconstructed with 3.0 mm slice thickness at 2.0 mm slice increment, with soft tissue kernel (I30) and a medium strength setting for SAFIRE. The chest and the abdominopelvic portions of the exam were reconstructed in both sagittal and coronal planes as well. The chest and the abdominopelvic acquisitions are done at fixed 120kVP, with Quality reference mAs of 240.

### Body composition measurements

**Fig. 1:**
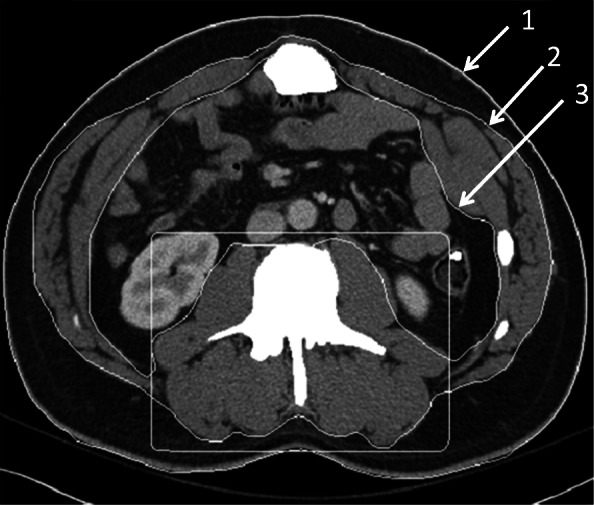
Example of segmentation of a computed tomography image with boundary lines between external air and subcutaneous fat (1), between subcutaneous fat and abdominal wall/paraspinal muscles (2), and between abdominal wall/paraspinal muscles and visceral fat (3). The box around the paraspinal muscles allows the algorithm to separately calculate the paraspinal muscle area (if this information is desired)

**Fig. 2:**
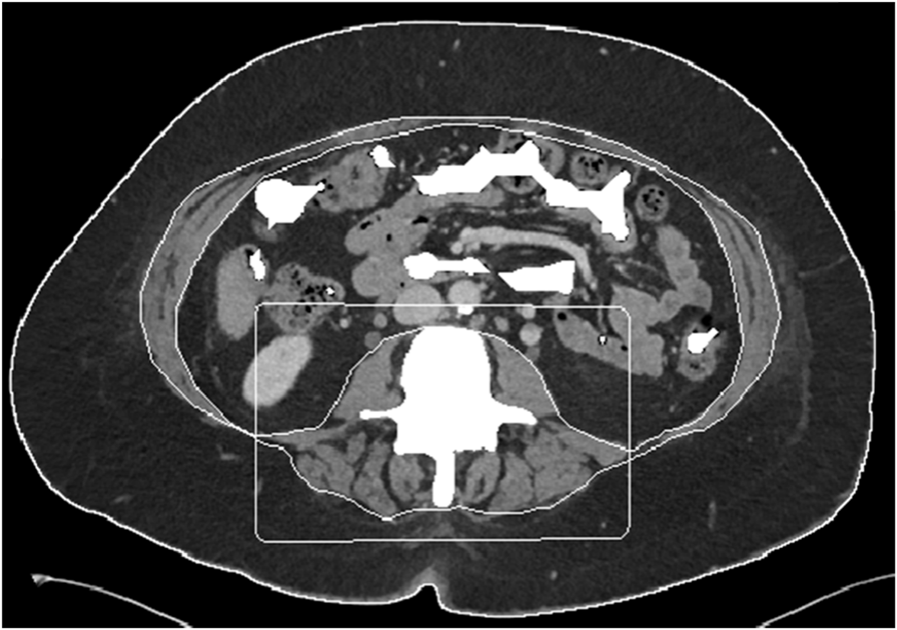
Segmentation of a computed tomography image of a 57-year-old sarcopenic female with a skeletal muscle index of 33 cm^2^/m^2^

**Fig. 3:**
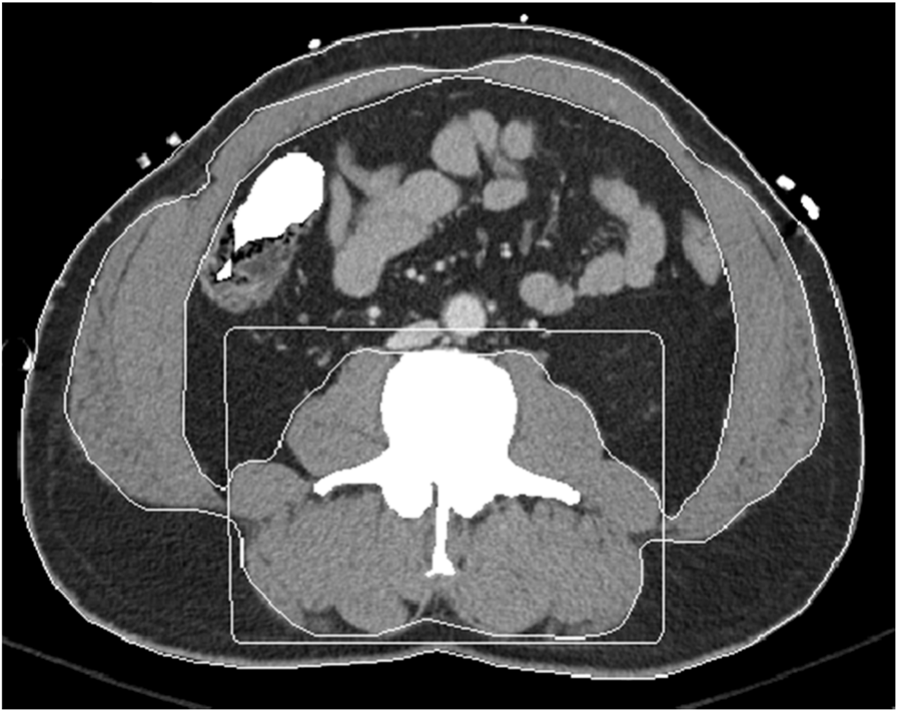
Segmentation of a computed tomography image of a 55-year-old nonsarcopenic male with a skeletal muscle index of 72 cm^2^/m^2^

One board-certified radiologist (DAD) and one radiology fellow (FIB) examined the admission CT scan and identified the single axial image at the level of the third lumbar vertebrae on which both transverse processes were fully visualized. These images were then analyzed in a semiautomatic process using the software BodyCompSlicer (developed at Mayo Clinic, Rochester, MN) [24]. BodyCompSlicer automatically placed three boundary lines between external air and subcutaneous fat, between subcutaneous fat and abdominal wall/paraspinal muscles, and between abdominal wall/paraspinal muscles and visceral fat. An example of a CT slice with boundary lines is included in Fig. 1. The reviewers then carefully inspected the boundaries and manually corrected the boundaries when necessary. The following muscles were outlined: psoas, erector spinae, quadratus lumborum, and the abdominal wall (including rectus abdominis, transverse abdominis, and internal and external oblique muscles). The software then calculated the skeletal muscle area, the area containing pixels between boundaries two and three excluding the spine, and having a CT attenuation value of -30 to 150 HU. The skeletal muscle area was then divided by the height of the patient squared (m^2^) to calculate the skeletal muscle index (SMI). Patients were categorized as sarcopenic according to international gender-specific consensus definitions (SMI = two standard deviations or less below the mean for healthy young adults aged 20–40), with male SMI lower than 55 cm^2^/m^2^ and female SMI lower than 39 cm^2^/m^2^ [25]. Figures 2 and 3 demonstrate CT slices of a sarcopenic patient and a nonsarcopenic patient.

### Data collection

Clinicopathological variables were collected which included age, sex, height, weight, body mass index, admission date, mechanism of injury, operating room date, time from admission to surgery, duration of operation, number of ribs plated, and number of plates used. The presence/absence of smoking history, chronic obstructive pulmonary disease, other sustained fractures, hemothorax, pneumothorax, chest tube placement, pneumonia, tracheostomy, and flail chest were recorded. Furthermore, the number of ribs fractured, number of total fractures, Rib score [26], ICU length of stay, total hospital length of stay, and the duration (days) of mechanical ventilation were documented. The Blunt Pulmonary Contusion-18 score [27], which is calculated by quantifying the degree of pulmonary contusion for each of the three zones in both lungs on a scale of zero to three, and modified frailty index, which has been proven to adequately reflect frailty and predict mortality and morbidity [28], were documented.

### Statistical analysis

Comparisons of patient demographics and clinical characteristics of patients with and without sarcopenia were made using *t* test, *χ*^2^ test, and Fisher’s exact test. Odds ratio and 95% CI were calculated. Multivariable logistic regression models were used to examine sarcopenia as a predictor of length of ICU and hospital stay, adjusted for age, body mass index, gender, and number of ribs fractured. Analyses were conducted using SAS (version 9.4; Cary, NC).

## Results

A total of 277 patients that sustained traumatic rib fractures underwent SSRF between 2009 and 2017. After exclusion criteria, the cohort consisted of 238 patients. Among the study population, 160 (67.2%) were male. The age was 60.4 ± 17.4 years (mean ± standard deviation). Considering the total of 238 patients included, the mechanism of initial injury was as follows: 104 (43.7%) automobile involved accidents (99 motor vehicle/motorcycle collisions, four automobile-pedestrian collisions, and one automobile-bike/ski collision), 90 (37.8%) falls, and 44 (18.5%) miscellaneous incidents. The miscellaneous category refers to mechanisms that do not occur with enough frequency to fall into a separate category. A few examples include penetrating injury secondary to farm equipment, explosion/blast injury, pedestrian-train collision, and crush injury. Out of the 104 patients involved in automobile accidents, 82 were nonsarcopenic, and 22 were sarcopenic. Out of the 90 patients involved in falls, 43 were nonsarcopenic and 47 were sarcopenic. Out of the 44 patients in the miscellaneous category, 25 were nonsarcopenic and 19 were sarcopenic.

Complete clinical, demographic, and injury characteristics of included patients are seen in Table 1. A total of 88/238 (36.9%) patients were classified as sarcopenic. Sarcopenic patients were older than nonsarcopenic patients, aged 69.1 ± 16.3 years (mean ± SD) *versus* 55.3 ± 16.0 years (*p* < 0.001). Sarcopenic patients demonstrated a lower mean body mass index compared to nonsarcopenic patients (27.2 ± 5.6 kg/m^2^ *versus* 31.6 ± 5.7 kg/m^2^, *p* < 0.001) and sarcopenic patients demonstrated significantly increased modified frailty index scores (1.5 ± 1.1 *versus* 0.9 ± 0.9, *p* < 0.001) but decreased Blunt Pulmonary Contusion-18 scores (1.2 ± 1.3 *versus* 1.8 ± 1.4, *p* < 0.001) compared to nonsarcopenic patients.

**Table 1:** Clinical, demographic, and injury characteristics

	Nonsarcopenic	Sarcopenic	Total	*p* value
**Gender**				0.025
Female	57 (38.0%)	21 (23.9%)	78 (32.8%)	
Male	93 (62.0%)	67 (76.1%)	160 (67.2%)	
**Age (years)**	55.3 (16.0)	69.1 (16.3)	60.4 (17.4)	< 0.001
**Body mass index (kg/m**^**2**^**)**	31.6 (5.7)	27.5 (5.0)	30.1 (5.8)	< 0.001
**Hospital length of stay (days)**	12.9 (8.2)	13.3 (7.2)	13.0 (7.8)	0.183
**Intensive care unit length of stay (days)**	4.9 (5.7)	5.9 (6.5)	5.3 (6.0)	0.146
**Days of mechanical ventilation**	3.1 (4.3)	2.8 (4.9)	3.0 (4.5)	0.304
**Modified frailty index 5**	0.9 (0.9)	1.5 (1.1)	1.1 (1.0)	< 0.001
**Rib Score**	2.5 (1.8)	2.2 (1.7)	2.4 (1.8)	0.279
**Blunt Pulmonary Contusion-18 score**	1.8 (1.4)	1.2 (1.3)	1.6 (1.4)	< 0.001
**Number of ribs fractured**	8.0 (4.0)	7.8 (4.3)	7.9 (4.1)	0.697
**Number of total fractures**	12.1 (7.0)	11.4 (6.4)	11.8 (6.8)	0.427
**Days to surgical stabilization of rib fractures**	4.4 (3.6)	3.8 (1.9)	4.2 (3.1)	0.292
**Flail chest**				0.158
No	51 (34.0%)	38 (43.2%)	89 (37.4%)	
Yes	99 (66.0%)	50 (56.8%)	149 (62.6%)	
**Pneumothorax**				0.016
No	45 (30.0%)	40 (45.5%)	85 (35.7%)	
Yes	105 (70%)	48 (54.5%)	153 (64.3%)	
**Hemothorax**				0.610
No	87 (58.0%)	54 (61.4%)	141 (59.2%)	
Yes	63 (42.0%)	34 (38.6%)	97 (40.8%)	
**Smoking**				0.207
No	114 (76.0%)	73 (83.0%)	187 (78.6%)	
Yes	36 (24.0%)	15 (17.0%)	51 (21.4%)	
**Chronic obstructive pulmonary disease**				0.006
No	140 (93.3%)	72 (81.8%)	212 (89.1%)	
Yes	10 (6.7%)	16 (18.2%)	26 (10.9%)	
**Tracheostomy placement**				0.623
No	139 (92.7%)	83 (94.3%)	222 (93.3%)	
Yes	11 (7.3%)	5 (5.7%)	16 (6.7%)	
**Pneumonia**				0.743
No	129 (86.0%)	77 (87.5%)	206 (86.6%)	
Yes	21 (14.0%)	11 (12.5%)	32 (13.4%)	
**Mechanism**				< 0.001
Auto-involved	82 (54.7%)	22 (25.0%)	104 (43.7%)	
Fall	43 (28.7%)	47 (53.4%)	90 (37.8%)	
Miscellaneous	25 (16.7%)	19 (21.6%)	44 (18.5%)	

Data are mean (standard deviation) or frequencies

Comparison of those with and without sarcopenia demonstrated no significant difference in duration of ICU stay (5.9 ± 6.5 *versus* 4.9 ± 5.7 days, *p* = 0.146) or duration of total hospital stay (13.3 ± 7.2 *versus* 12.9 ± 0.2 days, *p* = 0.183). The number of days of mechanical ventilation was not significantly different in those with or without sarcopenia (2.8 ± 4.9 *versus* 3.1 ± 4.3 days, *p* = 0.304). A total of 11/88 (12.5%) sarcopenic patients and 21/150 (14.0%) nonsarcopenic patients developed pneumonia (*p* = 0.743), while 5/88 (5.7%) sarcopenic and 11/150 (7.3%) nonsarcopenic patients received a tracheostomy (*p* = 0.623) Out of the 149 patients with flail chest, 50 (33.6%) were sarcopenic and 99 (66.4%) were nonsarcopenic (*p* = 0.158). There was no significant difference in the numbers of ribs fractured between those with and without sarcopenia (7.8 ± 4.3 *versus* 8.0 ± 4.0, *p* = 0.697). Furthermore, there was no significant difference in the time to SSRF between those with and without sarcopenia (3.8 ± 1.9 *versus* 4.4 ± 3.6 days, *p* = 0.292). The number of ribs fractured was a significant predictor of hospital (*p* < 0.001) and ICU length of stay (*p* = 0.003).

A total of four patients died while in the hospital after a prolonged duration of total hospital stay (range 10–20 days) and ICU stay (range 7–17 days) and a number of days of mechanical ventilation (range 4–10 days), all of them being sarcopenic. Fisher’s exact test demonstrated a *p* value of 0.018, with an odds ratio of 1.048 (95% confidence interval 1.000–1.010), revealing that sarcopenic patients in the cohort had a significant increased mortality risk when compared to nonsarcopenic patients.

## Discussion

To our knowledge, this is the first study to explore whether sarcopenia affected short-term outcomes in patients who underwent SSRF after sustaining traumatic rib fractures. In our cohort, there was no statistical significant difference in duration of stay in the ICU, total duration of stay in the hospital, and duration of mechanical ventilation in sarcopenic patients when compared to nonsarcopenic patients. These results are in contradiction to prior studies in the emergency surgery and trauma population, which have demonstrated that patients with sarcopenia have longer durations of stay in the ICU and hospital as well as a longer duration of mechanical ventilation [16, 23, 29].

However, sarcopenic patients did have a significant greater modified frailty index. The modified frailty index has been proven to reflect frailty and predict mortality and morbidity, identifying patients who are more susceptible to complications after surgical procedures [28]. Furthermore, sarcopenia has also been proven to negatively impact outcomes in many populations, including the surgical and trauma populations [16, 23, 30, 31]. Thus, it is interesting that the sarcopenic patients in our cohort had an increased modified frailty index, but did not have increased duration of stay in the ICU or hospital and did not have a longer duration of mechanical ventilation.

A total of four patients died while in the hospital, and all were sarcopenic. While our data did demonstrate that this was statistically significant (*p* = 0.018), the odds ratio [95% CI] was 1.048 [1.000, 1.010]; thus, this is likely of minimal clinical significance and death is nearly equally likely to occur in patients with sarcopenia and without sarcopenia. Even though more sarcopenic patients died in this cohort, this is not a novel and has been demonstrated on numerous prior studies, including studies focusing on patients undergoing emergency surgery and patients involved in trauma [16, 22, 23]. Additionally, all of these patients who died did have prolonged stays in the ICU and hospital with numerous days of mechanical ventilation. Thus, it is unclear if our data concerning duration of stay in the ICU and hospital as well as duration of mechanical ventilation when comparing sarcopenic and nonsarcopenic patients would have changed had all sarcopenic patients survived. However, we expect that the change in our data would have been minimal, as these patients had already spent considerable time in the ICU and hospital and on mechanical ventilation.

There was no statistical significance in the numbers of ribs fractured, the number of patients with flail chest, and the number of days between the trauma and the rib fixation surgery between patients with sarcopenia and patients without sarcopenia. However, the number of ribs fractured was a significant predictor of hospital and ICU length of stay. This confirms earlier results revealing that increasing number of fractured ribs is associated with greater morbidity and mortality [3].

There are limitations to this study. This is a single institution, retrospective review, and while our institution does have criteria for SSRF, there is a potential for selection bias in choosing patients suitable for an operation. Furthermore, since this study involved patients with traumatic rib fractures, many of them likely had concomitant injuries within the chest, abdomen, pelvis, brain, and spine which would play a role in their overall condition and ultimately their disposition.

In conclusion, sarcopenic patients in our cohort had statistically significant higher modified frailty indices, without longer hospital or ICU lengths of stay, or more days of mechanical ventilation. Thus, sarcopenia should not preclude patients with traumatic rib fractures from undergoing SSRF. We feel that this is quite important as numerous other studies have demonstrated that sarcopenic patients have worse outcomes. However, more research needs to be completed in regards to this topic before definitive conclusions are drawn. For example, a case-matched control study, and ultimately a randomized controlled study, comparing patients who underwent SSRF and those who did not would be important next steps in verifying our results.

## Data Availability

The datasets generated and/or analyzed during the current study are not publicly available due because the dataset is a private institutional data set but are available from the corresponding author on reasonable request.

## Abbreviations

CT: Computed tomography
ICU: Intensive care unit
SMI: Skeletal muscle index
SSRF: Surgical stabilization of rib fractures
